# Brute-force pharmacophore assessment and scoring with Open3DQSAR

**DOI:** 10.1186/1758-2946-3-S1-P39

**Published:** 2011-04-19

**Authors:** P Tosco, T Balle

**Affiliations:** 1 Department of Drug Science, University of Turin, Torino, Italy, 10125, Italy; 2 Department of Medicinal Chemistry, University of Copenhagen, København, Denmark, 2100, Denmark

## 

During the last year we have developed Open3DQSAR, an open-source tool aimed at high-throughput 3D-QSAR model building and evaluation [1]. During development, high performance and ease of automation were defined as primary goals; the first was achieved by implementation of parallelized algorithms and the second by adopting a scriptable interface.

Molecular interaction fields of different kinds (namely, built-inMMFF94 or GAFF steric/electrostatic fields, GRID fields and QM electron density/ESP maps) can be seamlessly computed and combined in a 3D-QSAR model from within Open3DQSAR thanks to its interoperability with OpenBabel, Molecular Discovery GRID and most QM packages.

**Figure 1:**
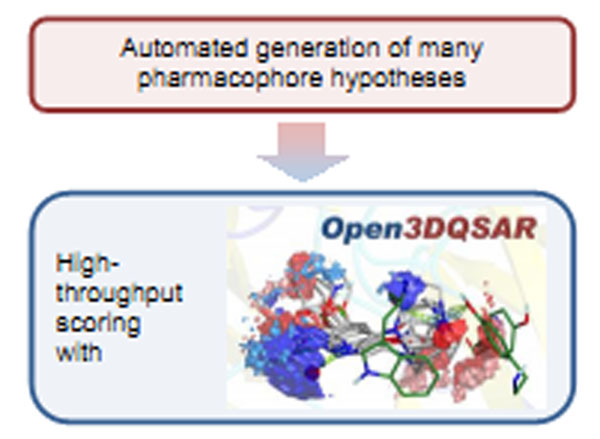
Proposed brute-force workflow.

In the poster, we describe the recent integration in Open3DQSAR of existing open-source tools to perform conformational analysis and molecular alignment, in order to automatically generate an exhaustive pool of pharmacophore hypotheses for a given dataset. Subsequently, such hypotheses are scored according to their ability to yield a predictive and robust 3D-QSAR model (Figure 1). We applied our brute-force pharmacophore assessment methodology on the benchmark datasets gathered by Sutherland et al. [2], to investigate whether the traditional alignment bottleneck in 3D-QSAR can be turned into a powerful instrument to challenge the goodness of a pharmacophore guess.
